# Serological and Molecular Epidemiology of Chikungunya Virus Infection in Vietnam, 2017–2019

**DOI:** 10.3390/v15102065

**Published:** 2023-10-08

**Authors:** Thanh Vu Nguyen, Mya Myat Ngwe Tun, Minh Thang Cao, Huy Manh Dao, Chan Quang Luong, Thi Kim Loan Huynh, Thi Thanh Thuong Nguyen, Thi Nhu Dao Hoang, Kouichi Morita, Thi Quynh Mai Le, Quang Duy Pham, Yuki Takamatsu, Futoshi Hasebe

**Affiliations:** 1 Department of Virology, Institute of Tropical Medicine, Nagasaki University, Nagasaki 852-8523, Japan; thanhvu.pasteur@gmail.com (T.V.N.); moritak@nagasaki-u.ac.jp (K.M.); 2 Graduate School of Biomedical Sciences, Nagasaki University, Nagasaki 852-8523, Japan; 3 Pasteur Institute in Ho Chi Minh City, Ho Chi Minh City 700000, Vietnam; tminhcao@gmail.com (M.T.C.); daohuymanh@gmail.com (H.M.D.); lcq33new@gmail.com (C.Q.L.); loanphuch64@gmail.com (T.K.L.H.); ntthanhthuong@gmail.com (T.T.T.N.); hoangnhudao74@gmail.com (T.N.D.H.); duyquang.pham@gmail.com (Q.D.P.); 4 DEJIMA Infectious Disease Research Alliance, Nagasaki University, Nagasaki 852-8523, Japan; 5 Center for Vaccines and Therapeutic Antibodies for Emerging Infectious Diseases, Shimane University, Izumo 690-8504, Japan; 6 National Institute of Hygiene and Epidemiology, Hanoi 100000, Vietnam; lom9@hotmail.com; 7 Vietnam Research Station, Institute of Tropical Medicine, Nagasaki University, Nagasaki 852-8523, Japan

**Keywords:** chikungunya, Vietnam, seroprevalence, molecular epidemiology

## Abstract

Chikungunya fever is an acute febrile illness caused by the chikungunya virus (CHIKV), which is transmitted by *Aedes* mosquitoes. Since 1965, only a few studies with limited scope have been conducted on CHIKV in Vietnam. Thus, this study aimed to determine the seroprevalence and molecular epidemiology of CHIKV infection among febrile patients in Vietnam from 2017 to 2019. A total of 1063 serum samples from 31 provinces were collected and tested for anti-CHIKV IgM and IgG ELISA. The 50% focus reduction neutralization test (FRNT_50_) was used to confirm CHIKV-neutralizing antibodies. Quantitative real-time RT–PCR (RT–qPCR) was performed to confirm the presence of the CHIKV genome. The results showed that 15.9% (169/1063) of the patients had anti-CHIKV IgM antibodies, 20.1% (214/1063) had anti-CHIKV IgG antibodies, 10.4% (111/1063) had CHIKV-neutralizing antibodies, and 27.7% (130/469) of the samples were positive in RT–qPCR analysis. The E1 CHIKV genome sequences were detected among the positive RT–qPCR samples. Our identified sequences belonged to the East/Central/South/African (ECSA) genotype, which has been prevalent in Vietnam previously, suggesting CHIKV has been maintained and is endemic in Vietnam. This study demonstrates a high prevalence of CHIKV infection in Vietnam and calls for an annual surveillance program to understand its impact.

## 1. Introduction

Chikungunya fever is an infectious disease caused by chikungunya virus (CHIKV)-infected *Aedes* mosquitoes [1,2]. The clinical manifestations of CHIKV infection are fever, rash, and especially polyarthralgia/polyarthritis, which can last from weeks to months [3]. This can lead to a misdiagnosis of CHIIKV infection as dengue. Early and accurate CHIKV infection diagnoses can contribute to a decrease in the disease burden in terms of the economy, society, and quality of life [4]. There are currently no effective antiviral treatments or vaccines for CHIKV infection [5,6]. Frequently employed diagnostic measures are real-time reverse transcription-polymerase chain reaction (RT–PCR) for CHIKV RNA detection and IgM antibody tests targeting CHIKV antigens [7,8,9]. CHIKV is a positive single-strand RNA virus of the *Alphavirus* genus in the *Togaviridae* family. Its genome encodes three structural proteins (capsid, envelope, and membrane) and five non-structural proteins (NSP1–5). CHIKV is categorized into three predominant genotypes: West African, Asian, and East/Central/South/Africa (ECSA), based on the envelope protein-coding genome sequence [10,11].

CHIKV was first identified in Tanzania in 1952 [12]. CHIKV was primarily sporadic in Asia with the Asian genotype [13,14,15,16,17]. After the ECSA genotype was detected in Kenya during 2004–2005, the virus pervasively infiltrated to Asia [18,19] and remains persistent in countries including India, Sri Lanka, Singapore, Malaysia, Thailand, and the Philippines [20,21,22,23,24,25,26,27]. From 2014–2015, the Asian and ECSA genotype of CHIKV caused major outbreaks in America, where it continues to be an important public health concern [28,29].

CHIKV was first identified in Vietnam in the 1960s [30], but there is limited information on the epidemiology, clinical, and molecular analysis of the virus in the country. Only sporadic investigations have been conducted over the decades, revealing intermittent CHIKV detections. An analysis of serum samples collected from October 2010 to December 2014 in southern Vietnam identified a seroprevalence of 0.07% (4 out of 5617 cases); four of the CHIKV isolates belonged to the Indian Ocean Lineage (IOL) within the ECSA genotype and were closely related to the 2011 Cambodian isolates [31]. Two instances of CHIKV were found in *Aedes aegypti* mosquitoes from Dac Nong and Long An provinces in surveillance conducted on 1104 adult mosquitoes and 12,041 larvae from September 2012 to September 2014 in five of the northern, central and southern provinces of Vietnam. However, that study could not confirm any CHIKV infection among 558 acute febrile patients [32]. Likewise, another study reported no CHIKV cases from 2012–2013 in one southern province [33]. Due to the scarcity of CHIKV data in Vietnam, we conducted this study to understand the situation of CHIKV infection in Vietnam. Our study may encourage future research endeavors to evaluate CHIKV infection in Vietnam.

## 2. Materials and Methods

### 2.1. Sample Population and Study Methodology

**Figure 1:**
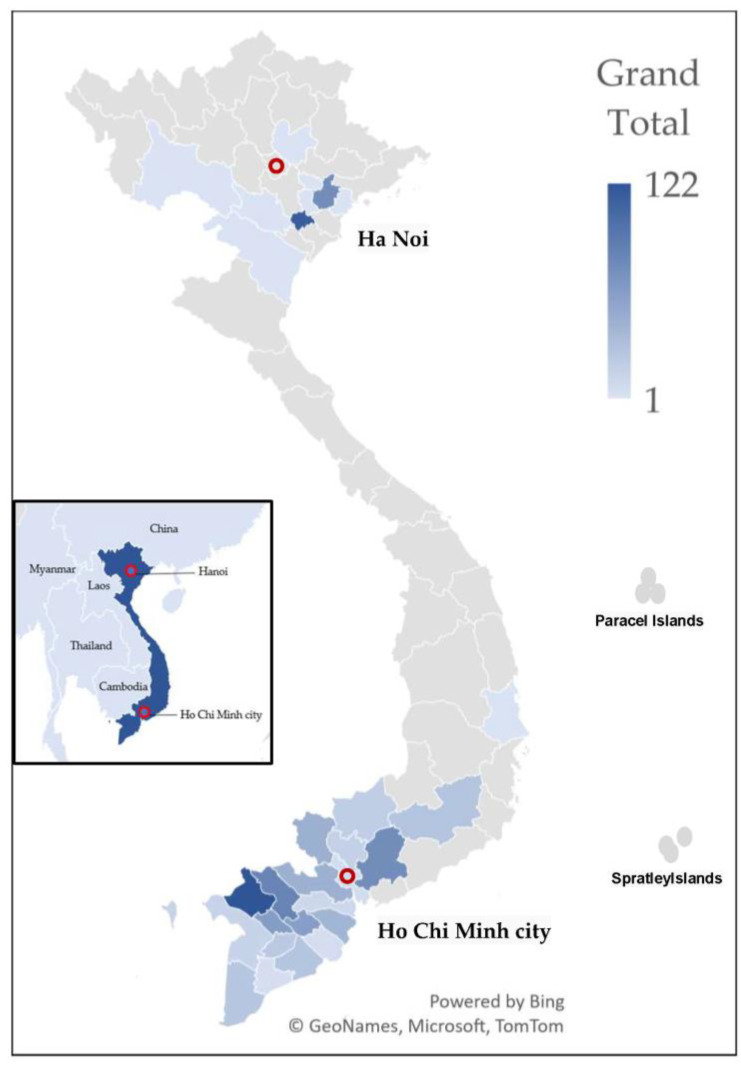
Sample distribution map. The inset map depicts the location of Vietnam and neighboring countries. This map illustrates the geographical distribution of serum samples collected from febrile patients in 21 southern provinces and 10 northern Vietnamese provinces. Dark blue indicates provinces with higher sample collection, and light blue represents with lower sample collection. The number of samples ranged from 1 to 122, with a median of 23.

The serum samples used in this study were obtained from leftover serum collected acute febrile patients as part of the dengue surveillance program conducted by the Pasteur Institute in Ho Chi Minh City and the National Institute of Hygiene and Epidemiology (NIHE) in Vietnam from 2017 to 2019. We randomly collected 1063 serum samples from these residual samples, distributed evenly across 2017, 2018, and 2019. These patients resided in 31 out of 63 Vietnamese provinces, primarily in Northern (10 provinces) and Southern Vietnam (21 provinces) (Figure 1 and Supplementary Table S1). The selection of patients during the sampling process was based solely on the following criteria: sample volume, quality of sample storage, and basic demographic information. Dengue test results were not considered in the sampling process.

### 2.2. Viruses and Cell Lines

This study used the CHIKV S-27 strain (African prototype) as an antigen for CHIKV IgM and IgG detection by Enzyme Linked Immunosorbent Assay (ELISA), and the neutralization test. C6/36 mosquito cells, grown in Eagle’s minimum essential medium (EMEM) supplemented with 10% fetal calf serum (FCS) and 50 U/mL penicillin and 50 μg/mL of streptmycine (P/S), were used for virus propagation at 28 °C. Vero cells (African green monkey kidney epithelial cells, ATCC CCL-81) grown in similar medium with C6/36 cells, were used for viral titration and neutralization tests at 37 °C with 5% CO_2_.

### 2.3. ELISA Identification of Anti-CHIKV IgM Antibodies

To detect anti-CHIKV IgM antibodies, we used an in-house IgM capture ELISA system [34,35,36,37,38,39,40]. The procedure began by coating the wells of 96-well microplates (Nalge Nunc International, Roskilde, Denmark), except for the blank wells, with an anti-human IgM goat IgG (Cappel ICN Pharmaceuticals, Aurora, OH, USA) in a coating buffer (pH 9.6). After overnight incubation and blocking with BlockAce (Yukijirushi Co, Tokyo, Japan), the samples and controls were diluted 1:100 in a solution of phosphate-buffered saline in Tween 20 (PBS-T) containing 10% BlockAce. The diluted samples were dispensed into duplicate wells. Subsequently, the CHIKV antigen was used as an assay antigen and incubated at 37 °C for 1 h. The wells were then incubated with a 1:400 dilution of Horseradish peroxidase (HRP)-conjugated anti-CHIKV mouse-derived recombinant E1 monoclonal antibody at 37 °C for 1 h. Staining was achieved by adding o-phenylenediamine dihydrochloride (OPD) (Sigma Chemical, St. Louis, MO, USA) solution and 0.03% hydrogen peroxide in a substrate buffer at pH 5.0. The reaction was terminated by adding 1 N sulfuric acid followed by incubation for 20–30 min at room temperature. The optical density (OD) was subsequently determined at 492 nm using a Multiskan Sky microplate spectrophotometer. A positive OD/negative control OD (P/N) ratio of ≥2.0 indicated a positive sample.

### 2.4. ELISA Identification of Anti-CHIKV IgG Antibodies

The presence of anti-CHIKV IgG antibodies were detected using an in-house indirect IgG ELISA protocol that was adapted from previous studies [34,35,36,37,38,39,40]. The 96-well microplates were coated with purified structural protein as an antigen in a coating buffer and incubated overnight at 4 °C. The wells were then incubated with samples from the study and control groups in duplicate wells at a dilution ratio 1:1000 in a solution of PBS-T with 10% BlockAce. HRP-conjugated anti-human IgG (American Qualex, San Clemente, CA, USA) was added to the wells at a dilution ratio 1:20,000. The wells were then labeled with OPD, and the OD was measured at 492 nm using the Multiskan Sky instrument. The IgG titers in serum samples were determined based on a standard curve, and a threshold titers of ≥3000 was classified as IgG-positive. 

### 2.5. Detection of Neutralization Antibody against CHIKV by FRNT_50_

The 50% focus reduction neutralization test (FRNT_50_) assay was used to validate the neutralizing activity of antibodies in seropositive individuals, as in previous studies [34,36,37,38]. The heat-treated serums were combined with 40 focus-forming units (FFU) in equal volumes and incubated for 1 h at 37 °C with 5% CO_2_. The mixture was then evenly distributed to duplicate wells in 96-well plates containing Vero cells and incubated for 1 h at 37 °C. Subsequently, 150 μL of a maintenance medium comprising 2% fetal calf serum (FCS) and 1.25% methylcellulose 4000 (Wako, Osaka, Japan) in EMEM were applied to the wells and incubated for 24 h at 37 °C. The cells were fixed with a 4% paraformaldehyde solution and permeabilized by Nonidet P-40 (Nacalai Tesque, Kyoto, Japan). The cells were then immunostained using anti-CHIKV rabbit IgG (developed in-house) at a 1:2000 dilution and incubated for 1 h at 37 °C. Afterward, the cells were treated with HRP-conjugated goat anti-rabbit IgG (American Qualex, San Clemente, CA, USA) at a 1:2000 dilution and incubated for 1 h at 37 °C. The FRNT_50_ titer was determined as endpoint serum dilution that exhibited a ≥50% reduction over the mean number of foci in control wells. Samples with a neutralization titer of 10 or higher were classified as positive.

### 2.6. CHIKV Genome Detection Using Real-Time Quantitative PCR (RT–qPCR)

RNA was isolated from serum samples using the QIAamp Viral RNA Mini Kit (QIAGEN, Hilden, Germany) following the manufacturer’s instructions. Subsequently, reverse transcription was performed on the RNA sample using the PrimeScript RT Kit (Takara Bio, Shiga, Japan) to create cDNA. To identify the genome of CHIKV, a SYBR green quantitative RT–PCR test (Takara Bio, Shiga, Japan) was used, specifically targeting the envelope protein 1 (E1-129 bp) and non-structure protein 2 (NSP2-107 bp). The primer set used for amplifying E1 and NSP2 in quantitative PCR has been previously documented in the scientific literature [41,42,43] and can be found in Supplementary Table S2. A standard curve was plotted using CHIKV RNA obtained from a CHIKV S-27 strain. The curve comprised seven dilutions spanning a range of 10^1^ to 10^7^ copies/mL. The estimated detection limit for this assay was approximately 10^2^ copies/mL.

### 2.7. Genomic Characterization of CHIKV

The ReverTra Ace kit (Toyobo, Osaka, Japan) was used for reverse transcription of the RNA samples that tested positive for CHIKV with real-time RT–PCR. Then, the E1 gene segment (294 bp) was amplified using the PrimeSTAR kit (Takara Bio, Shiga, Japan), and the amplicon DNA sequence was acquired by applying the Sanger method. The primers used [41] are documented in Supplementary Table S2. The nucleotide sequences were analysed using DNADynamo v. 1.63 (Blue Tractor Software). Then, the sequences were aligned with CHIKV global sequences using MAFFT v. 7.520 [44] and subjected to phylogenetic analysis using the maximum-likelihood method with 1000 bootstrap replicates in MEGA 11 [45,46]. The Tamura–Nei and invariant site models were employed for this analysis after finding the best-fit model based on the Bayesian information criterion (BIC) using W–IQ–TREE [47,48,49,50]. The nucleotide sequences obtained from current study were submitted to the GenBank database under accession numbers OR492236, OR492237, OR492238, OR492239, OR492240, OR492241, and OR492242.

### 2.8. Data Analysis

Research data were cleaned using Microsoft Excel (2019, v. 1808). The data were then analyzed using three statistical software packages: Microsoft Excel, GraphPad Prism 10.0.1, and Stata 16. Chi-square tests and generalized linear models were used to determine the differences in the proportions of risk factors among the groups. ANOVA, the Kruskal–Wallis H test, and the Mann–Whitney U tests were used to determine the difference in medians among the groups. Dunn’s test and Bonferroni’s correction method were used to determine which group means were significantly different from each other. Pearson correlation coefficients were used to assess the correlation between the neutralization antibody titer and the P/N ratio of the IgM and IgG titers. All results were considered statistically significant at *p* < 0.05.

## 3. Results

### 3.1. Demographic Characteristics

**Figure 2:**
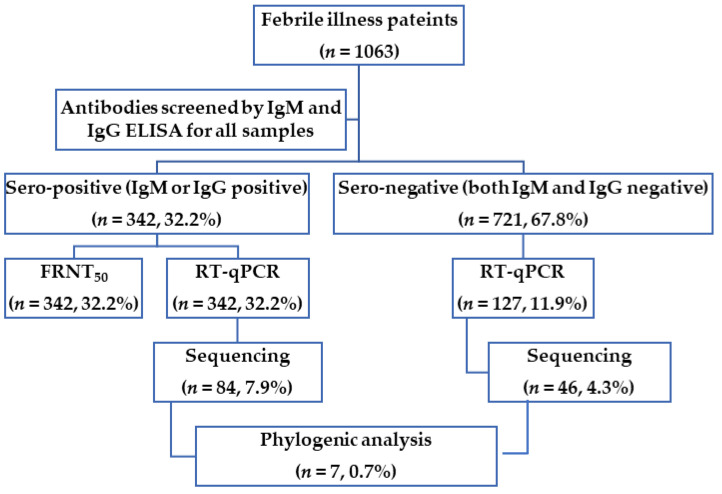
Flowchart and analyses performed in this study. The graph shows the research flowchart and the number of serum samples used for major tests and analysis in this study.

Figure 2 demonstrated the flow chart of this study and the number and proportions of positive samples were summarized. We screened 1063 serum samples from febrile illness patients for anti-CHIKV IgM and IgG antibodies ELISA, followed by FRNT_50_ for seropositive samples. The RT–qPCR was then used to measure the amount of CHIKV RNA in all samples. However, only 127 seronegative samples with sufficient serum volume were used to quantify CHIKV RNA by RT–qPCR. Finally, all positive samples for the CHIKV RNA genome were sequenced to construct phylogenetic trees.

We summarized the demographic characteristics of the study population (Table 1). The study sample was evenly distributed across the years 2017 (30.7%), 2018 (30.7%), and 2019 (34.6%). The gender distribution of the study population was 44.2% female and 55.8% male. Most of the patients (73.4%) were from the southern provinces of Vietnam, which are known for their high prevalence of mosquito-borne diseases such as dengue. The remaining patients (26.6%) were from the northern provinces of Vietnam. The patients were categorized into four age-groups based on their risk of severe CHIKV disease including ≤5 years, 6–15 years, 16–45 years, and 46 years and older. As shown in Table 1, the 6 to 15-year-olds and 16 to 45-year-olds had the highest proportions, accounting for 36.9% and 34.8% of the patients, respectively. Most patients (66%) were initially diagnosed with either dengue or suspected dengue, while a smaller proportion (34%) was diagnosed with non-specific febrile illness.

### 3.2. Prevalence of Anti–IgM and Anti–IgG CHIKV Antibodies in the Study Population

As shown in Table 2, the prevalence of anti-CHIKV IgM and IgG antibodies were 15.9% (169/1063) and 20.1% (214/1063), respectively. In addition, 3.9% (41/1063) of the patients had both anti-IgM and anti-IgG CHIKV antibodies. Overall, 32.2% (342/1063) of the patients were seropositive for anti-CHIKV IgM and/or IgG antibodies. The prevalence of anti-CHIKV IgM antibodies was highest in 2018 (20.9%), whereas the anti-CHIKV IgG prevalence was highest in 2017 (23.6%). The overall seropositive rate for anti-CHIKV IgM and/or IgG antibodies remained similar in 2017, 2018, and 2019 (32.3%, 32.2%, and 32%, respectively). There was a significant difference in the seropositive rate for anti-CHIKV IgM and/or IgG antibodies between the south (35.9%) and the north (21.9%) of the country (*p* < 0.0001). There was no significant difference in the presence of anti-CHIKV IgM and/or IgG antibodies based on genders or clinical diagnoses. However, there was a significant difference in the presence of anti-CHIKV IgM and/or IgG antibodies across age groups (*p*-values of 0.012, 0.000, 0.009, and 0.000).

**Figure 3:**
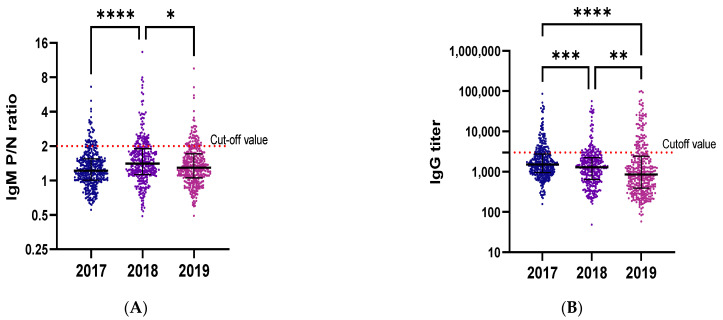

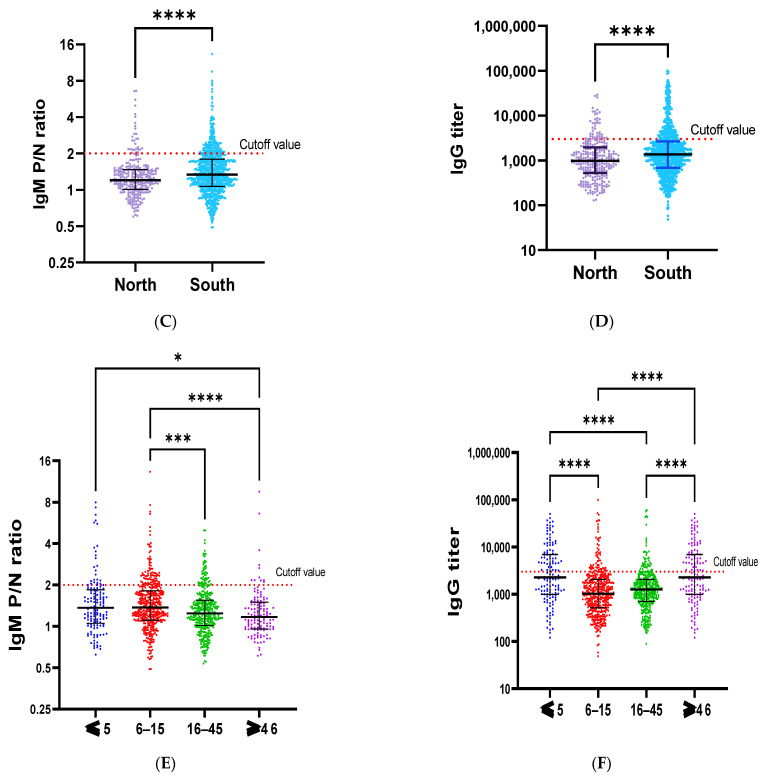
Distribution of IgM P/N ratios and IgG titers over the years, across regions, and age groups. Distribution of the IgM P/N ratio and IgG titer was investigated over the years (**A**,**B**), across the regions (**C**,**D**)**,** and across age groups (**E**,**F**). The P/N ratio is the ratio of the optical density (OD) of the sample to the OD of the negative control. The cutoff value is the red dot line with P/N ratio of 2 for IgM, and titer of 3000 for IgG. The samples with a titer or P/N ratio higher than the cutoff value were considered as positive. *p* value is defined as follows * *p* ≤ 0.05; ** *p* ≤ 0.01; t *** *p* < 0.001 and **** *p* < 0.0001.

To understand the characteristics of CHIKV seroprevalence, we analyzed the IgM/IgG status in detail (Figure 3). Regarding the year of illness, the mean P/N ratio of IgM was the highest in 2018, followed by 2019 and 2017, whereas the mean titer of IgG was the highest in 2017, followed by 2018, and the lowest was in 2019. Interestingly, the mean of both IgM P/N ratios and IgG titers were higher in the south than in the north. Regarding the age groups, the mean P/N ratios of IgM was the highest in those under 5, followed by those aged 6 to 15 years, 16 to 45 years, and the lowest was in the over-46 group. The over-46 group had the highest mean titer of IgG, followed by those under 5, 16 to 45 years, and 6 to 15 years.

### 3.3. Prevalence of Anti-CHIKV Neutralizing Antibodies (NAbs) in This Study

Table 3 shows the activity of the neutralizing antibodies against CHIKV. Patients with a neutralizing antibody titer of 10 or higher were considered to have anti-neutralizing activity against CHIKV. The proportion of patients with neutralizing antibodies is described by gender, age groups, regions, years, and clinical diagnoses at the time of sample collection. A statistically significant difference in the proportion of patients with neutralizing antibodies was found between age groups (*p* = 0.000).

**Figure 4:**
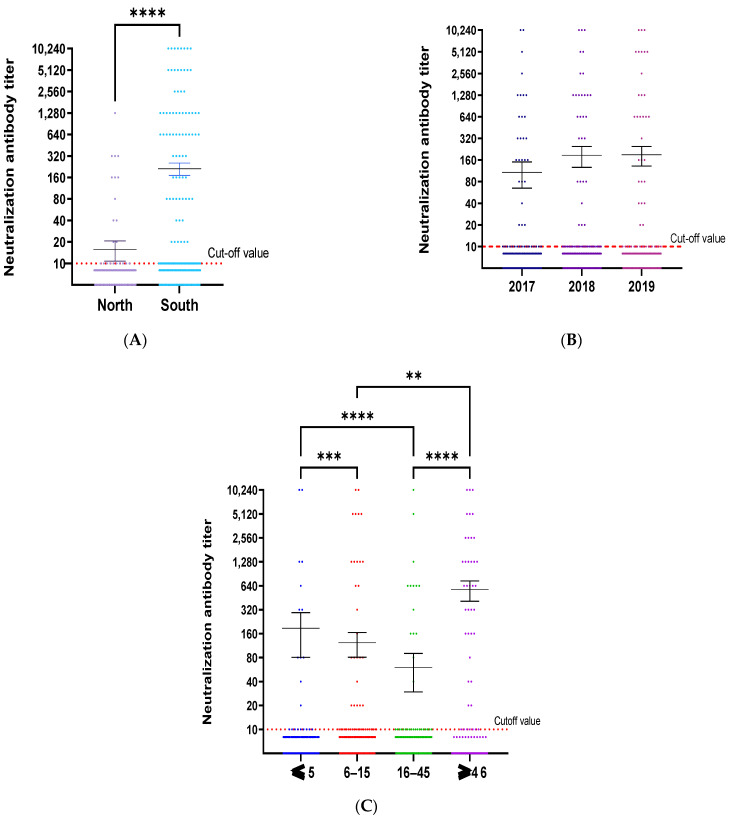
Neutralization titer distribution by regions, years and age groups. The distribution of neutralization titer by regions (**A**), years (**B**), and age groups (**C**) is shown. The cutoff value was defined as the red dot line corresponding to a value of 10 for neutralization antibody titer. The sample with a titer higher than the cutoff value was considered as positive. The mean of CHIKV-neutralizing antibodies was compared by regions, years, and age groups using the Kruskal–Wallis test, with the Dunn-Bonferroni correction method for multiple comparison tests. *p* value was defined as follows ** *p* ≤ 0.01; *** *p* < 0.001 and **** *p* < 0.0001.

To understand the characteristics of anti-CHIKV neutralizing antibodies, we analyzed the distribution of the neutralizing antibody titer by years, regions, age groups, and clinical diagnoses. Figure 4A showed that the activity of neutralizing anti-CHIKV antibodies in the south was significantly higher than in the north (*p* < 0.0001). There was no significant difference in neutralization titer over the years (Figure 4B). The highest mean NAbs titer was observed in the over-46-age group (Figure 4C). This finding is consistent with the results of further analyses of the mean NAbs titer in the age groups of each region (Figure S1A,B).

### 3.4. Correlation between CHIKV IgG and IgM Antibodies with Neutralizing Antibody (NAbs)

**Figure 5:**
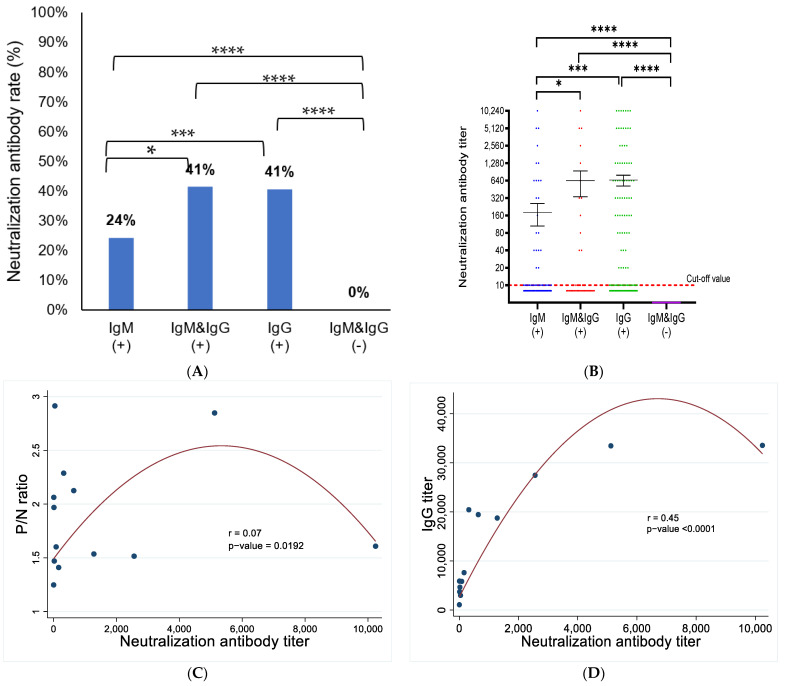
Presence of CHIVK neutralizing antibodies in seropositive groups. The presence of NAbs was compared between groups of patients that were positive for IgM and/or IgG, using the Chi-square test for each pair of groups (**A**). The mean of the NAbs was compared between groups of patients that were positive for IgM and/or IgG, using the Kruskal–Wallis test, with the Dunn-Bonferroni correction method for multiple comparison tests (**B**). The Pearson correlation coefficient was used to assess the correlation between NAbs titer and the P/N ratio of IgM (**C**) and the correlation between NAbs titer and IgG titer (**D**). *p* value was defined as follows * *p* ≤ 0.05; *** *p* < 0.001; **** *p* < 0.0001.

The presence of NAbs among seropositive patients is illustrated in Figure 5A. The proportion of patients with NAbs was significantly higher in the IgG-positive group (*p* < 0.0001) and both IgM/IgG positive groups (*p* = 0.013) than in the IgM-only-positive group. The distribution of the NAbs titer was also significantly different between the IgM-only-positive group and the IgG-only-positive group (*p* < 0.0001) and both IgM/IgG positive groups (*p* = 0.012). The titer and NAbs positive rate were both significantly higher in all seropositive groups than in the seronegative group, with *p*-values < 0.0001. The correlations between the NAbs titer and IgM P/N ratio (Figure 5C) and NAbs with IgG titer (Figure 5D) were evaluated using the Pearson correlation coefficient. A weak positive correlation was found between the NAbs titer and the IgM P/N ratio (r = 0.07, *p* < 0.05), and a moderate positive correlation was found between the NAbs titer and the IgG titer (r = 0.45, *p* < 0.0001).

### 3.5. CHIKV Genome Detection and Correlation between the CHIKV Genome and Antibodies

We intended to screen all samples for CHIKV RNA. However, because of the limited sample volume, we could only screen 469 (44.1%) samples, of which 342 were positive for IgM and/or IgG, and 127 were seronegative (negative for IgM and IgG). Of the 469 screened samples, 130 (27.7%) were positive for CHIKV RNA (Table 4). No significant differences were found in the CHIKV RNA detection rate between groups divided by gender, age groups, regions, year, and clinical diagnoses.

**Figure 6:**
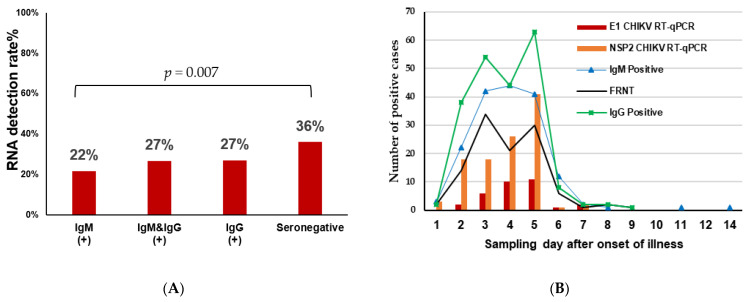
Comparison of CHIKV RNA presence in seroprevalence groups based on real-time PCR test results. The rates of RNA detection in seropositive and seronegative groups were compared pairwise using the Chi-square test (**A**). The relationship between the antibody and RNA detection time is depicted based on the days from the onset of symptoms (**B**).

Figure 6A shows that the presence of CHIKV RNA in the seronegative group (36%, 46/127) was significantly higher than in the IgM antibody-only group (22%, 37/169; *p* = 0.007). However, there was no significant difference between the seronegative group and the IgG antibody-only group (27%, 58/214; *p* = 0.077) and the group of both IgM and IgG positives (27%, 11/41, *p* = 0.269). Thus, these findings suggest that the number and rate of CHIKV RNA-positive patients may be higher than what was detected in this study.

Of the 130 patients with detectable CHIKV RNA, 83.8% (109 patients) were positive for RNA, using the NSP2 protein primer set, 24.6% (32 patients) were positive, using the E1 protein primer set, and 8.5% (11 patients) were positive, using both sets of primers. Further analysis in Figure 6B shows the relationship between the number of cases and the time of detection is shown in more detail.

### 3.6. CHIKV Sequence Analysis

**Figure 7:**
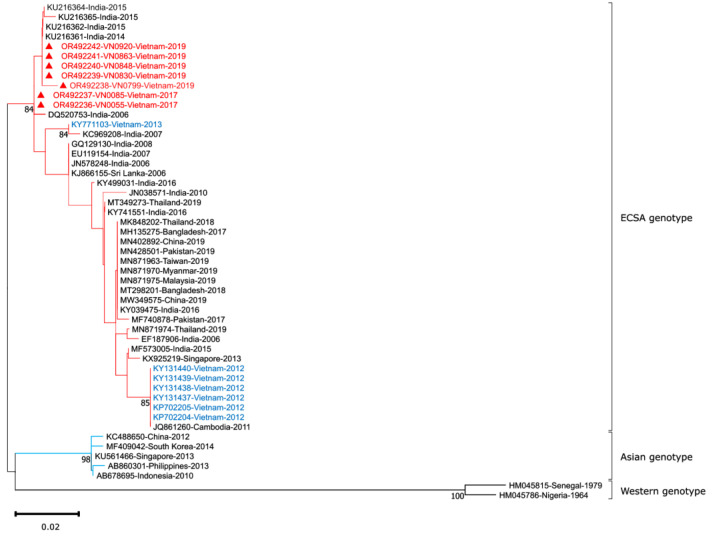
Evolutionary analysis by the maximum likelihood method. The evolutionary history of CHIKV was inferred using the maximum likelihood (ML) method and the Tamura–Nei model with 1000 bootstrap replications. Bootstrap confidence values are displayed at the branch nodes. The initial trees for the heuristic search were obtained automatically by applying the Neighbor–Join and BioNJ algorithms to a matrix of pairwise distances estimated using the Tamura–Nei model, and then selecting the topology with the superior log likelihood value. The rate variation model allows for some sites to be evolutionarily invariable. This analysis involved 51 nucleotide sequences, including partial E1 sequences from seven CHIKV isolates in this study, along with selected sequences from the GenBank database. The final dataset included 294 positions. Evolutionary analyses were conducted in MEGA 11. The three main CHIKV genotypes are highlighted in different colors, with isolates from the current study indicated by red triangles and red text. The sequences with blue text were CHIKV sequences from previous studies conducted in Vietnam.

To better understand the characteristics of the CHIKV genome in Vietnam, we amplified a fragment of the E1 gene (294 bp) from 130 RT–qPCR positive samples. The amplification products were then sequenced and analyzed with the global CHIKV strains by phylogenetic tree construction. We identified the presence of CHIKV E1 gene in 7 samples, and the phylogenetic analysis was conducted including these samples. The CHIKV isolates detected in current study were highly similar to the ECSA isolates from the major outbreaks in India from 2006–2008 and from 2014–2015 (99.3–100% nucleotide similarity), and the Vietnam CHIKV isolates detected in 2013 (98.6–98.9% nucleotide similarity). However, they were less similar to the Vietnam CHIKV isolates detected in 2012 (96.9–97.6% nucleotide similarity). This result demonstrates that the sequences found in this study were in the same lineage of previously detected ones in India and Vietnam (Figure 7). However, the limited sequence length analyzed in this study suggests that more data is needed to confirm this finding.

## 4. Discussion

This study is one of the largest-scale studies on CHIKV infection ever conducted in Vietnam. Samples were collected from 31 of the 63 provinces across the country from 2017–2019 for serological and molecular epidemiological analyses. Here, we balanced the number of samples over the years and collect samples from many places to increase the representativeness for the whole of Vietnam. We also focused on samples distributed in different regions, with particular attention to the southern part of the country, which is known for its high prevalence of mosquito-borne diseases such as dengue [51,52,53,54]. Therefore, the results of this study reflect a more representative situation of CHIKV infection in Vietnam. During the same period (2017–2019), we also observed CHIKV outbreaks in neighboring countries, including India (139,727 possible cases and 21,961 confirmed cases from 2018–2019) [55], Bangladesh (13,176 confirmed cases in 2017) [56], Thailand (approximately 15,000 confirmed cases from 2018–2019) [27], and Myanmar (confirmed the presence of CHIKV from 2018–2019) [35,37,39].

We found that 15.9% of patients were IgM-positive and 20.1% were IgG-positive. There have been variations in CHIKV seroprevalence in Vietnam in previous reports. For instance, 0% positivity for anti-CHIKV IgM and 50% positivity for anti-CHIKV IgG among 44 clinical dengue suspected cases were reported in 2006 [36]. In other studies, a positivity rate of 59.4% (17/32 acute febrile patients) was observed for anti-CHIKV IgM in one Vietnamese southern province from 2010–2011 [57], whereas 0.07–0.18% positivity for anti-CHIKV IgM was reported in few provinces in Vietnam [31,32,33]. Our results indicate a high prevalence of CHIKV infection in Vietnam. The proportion of patients with IgG was higher than the proportion of patients with IgM, and 16.3% (173/1063) of patients had only anti-CHIKV IgG antibodies, suggesting previous CHIKV exposure. As IgM and IgG against CHIKV are typically detectable 3–4 days and 6–7 days following onset of symptoms, respectively, some patients in the present study had both IgM and IgG at the same time.

Additionally, we demonstrated that the seropositive rate for anti-CHIKV IgM and/or IgG antibodies was significantly higher in the southern Vietnam (35.9%) than in the northern region (21.9%). This is consistent with the higher prevalence of mosquito-borne diseases in the southern part of the country [51,52,53,54]. Finally, we found that the higher IgG rate in children under 5 (45.6%) than in other age groups. This is likely due to the fact that children under 1 year old (57.6%) (Figure S2) are likely to have antibodies transferred from the mother [58,59]. However, the rate of IgG in the 1- to 5-year-old group is still high, suggesting that more research is needed to understand this condition. The highest proportion of patients with IgM antibodies was in the 6- to 15-year-old group (19.6%), followed by the under-5-year-old (17.6%), 16- to 45-year-old (13.4%), and over-46-year-old (9%) groups. This may be due to several factors, including the time of blood collection after disease onset and previous exposure to the virus [60,61,62,63,64,65,66,67,68,69]. Other factors that can affect the patient’s immune response include general health, nutritional status, and medication use. In the 6- to 15-year-old group, the highest proportion of patients had blood samples collected ≥4 days after disease onset (68%, 286/423), followed by the 16- to 45-year-old group (62%, 234/382), the under-5 group (57%, 77/136), and the over-46-year-old group (56%, 68/122). Future studies are needed to better understand the impact of these factors on the high IgM rate in the younger group.

The overall rate of anti-CHIKV neutralization antibodies in this study was 10.4% (111/1063). This value is lower than those reported in Myanmar in 2019 (18.9%) [70], 2013, 2015, and 2018 (32.5%) [37], but higher than those reported in Nepal (7.3%) [38] and Pakistan (6%) [71]. The neutralization activity of CHIKV was significantly higher in the south than in the north, which is consistent with the seroprevalence status. The high IgG status and high NAbs titer in the 46-years-old group compared to younger groups suggests that CHIKV has been present in Vietnam for long time. Further analysis of the NAbs titer across age groups and by regions confirmed this hypothesis (Figure S1). The NAbs titer was higher in the IgG-positive group than in the IgM-positive group, which is consistent with previous studies [37,72].

CHIKV viremia has been reported to be persist for up to 12 or 13 days after onset [73,74,75,76]. Among the tested 469 samples, 130 (27.7%) were positive in RT–qPCR analysis, including 84 (24.6%) of the 342 seropositive samples, and 46 (36%) of the 127 seronegative samples. The positivity of CHIKV RNA in seronegative samples is consistent with previous findings [40,75,77]. This suggests that the study’s RNA detection rate would likely be higher than the current results if sufficient volume had been available to test all seronegative samples. Among the 130 RT–qPCR positive samples, 109 (83.8%) were positive with the NSP2 primer set and 32 (24.6%) were positive with the E1 primer set. We hypothesize that two possible reasons for this discrepancy are: (1) a genetic mutation in E1 in some positive patients that reduces the ability to detect the E1 gene, and (2) low RNA concentration in some positive samples, possibly due to sample degradation from long-term storage or repeated freeze-thaw cycles. Although only a limited number of samples were subjected to genome detection because of the insufficient serum left, the high prevalence for CHIKV indicates the importance of this viral infection in Vietnam. We analyzed the relationship between the course of the disease and the presence of CHIKV by viral genome detection and host antibody positivity. Interestingly, 3–5 days after the onset seemed to be the best time for molecular diagnosis. Further analysis of the IgM and RNA detection rates in each region revealed that the presence of IgM and RNA gradually increased over the years in the southern region but may have gradually decreased in the northern region. However, these differences were not statistically significant (*p* < 0.05) (Figure S3).

By reading the sequence of the E1 gene in comparison to CHIKV strains deposited in Genbank, we found our sequences belongs to the ECSA genotype, which includes previously sequences detected in Vietnam. The sequences detected in the present study share a high degree of similarity (99.3–100% nucleotide similarity) with the Indian Ocean Lineage within ECSA genotype of the Indian isolates detected during the 2006 and 2014–2015 outbreaks. This suggests that the virus that caused the outbreaks in India in 2006 and 2014–2015 may be circulating in the study population. Through a comparison with outbreaks in neighboring countries during the same period (2017–2019), we found that the same CHIKV ECSA genotype caused outbreaks in India from 2018–2019 [55], Bangladesh in 2017 [56], Thailand from 2018–2019 [27], and Myanmar in 2019 [35,37,39], highlighting the importance of this genotype in Asian countries. The limited sequence length of current study hinders a comprehensive discussion. Thus, timing of sample collection and sample quality (degraded RNA) should be considered in viral genome sequencing.

## 5. Conclusions

Our study confirmed the high prevalence of the chikungunya virus (CHIKV) in Vietnam from 2017–2019. This finding provides a basis for further research to better understand the serological and molecular epidemiology of CHIKV, as well as the need for comprehensive and periodic surveillance of CHIKV in Vietnam in the future.

## Figures and Tables

**Table 1:** Demographics of the study population (*n* = 1063). Number of samples and proportion of samples for each factor (year of illness, regions, age group, and clinical diagnoses) from the entire study population and the population grouped by sex.

Variables	Overall (*n*, % ^+^)	Female (*n*, % ^++^)	Male (*n*, % ^++^)
**Year of illness**			
2017	368 (34.6)	157 (42.7)	211 (57.3)
2018	326 (30.7)	143 (43.9)	183 (56.1)
2019	369 (34.7)	170 (46.1)	199 (53.9)
**Regions**			
North	283 (26.6)	117 (41.3)	166 (58.7)
South	780 (73.4)	353 (45.3)	427 (54.7)
Age groups *			
≤5	125 (11.8)	46 (36.8)	79 (63.2)
6–15	392 (36.9)	171 (43.6)	221 (56.4)
16–45	370 (34.8)	168 (45.4)	202 (54.6)
≥46	176 (16.6)	85 (48.3)	91 (51.7)
Clinical diagnoses **			
Non-specific febrile illness	361 (34)	147 (40.7)	214 (59.3)
Suspected Dengue	58 (5.5)	23 (39.7)	35 (60.3)
Dengue without warning sign	604 (56.8)	280 (46.4)	324 (53.6)
Dengue with warning sign	26 (2.4)	10 (38.5)	16 (61.5)
Severe Dengue	14 (1.3)	10 (71.4)	4 (28.6)
**Overall**	1063	470 (44.2)	593 (55.8)

^+^ % in total for each variable. ^++^ % in each lane. * Age groups were defined based on the date and year of illness. ** Clinical diagnoses identified on patient’s initial hospital visit.

**Table 2:** Anti-CHIKV seroprevalence rate in the study. The proportion of patients with only IgM positive (1), only IgG positive (2), both IgM and IgG positive (3), and IgM positive and/or IgG positive (4) were compared by year, regions, genders, age group, and clinical diagnoses, using the Chi-square or Fisher’s exact test. If the difference was significant (*p* < 0.05) for any factor, it was further analyzed in more detail in the following sections.

Variable	*n* = 1063 (%) ^+^	IgM (+) *n*, (%) ^++^, (1)	IgG (+) *n*, (%) ^++^, (2)	IgM (+) and IgG (+) *n*, (%) ^++^, (3)	IgM (+) and/or IgG (+) *n*, (%) ^++^, (4)	*p*-Value (χ² or Fisher Test)
**Year of illness**						
2017	368 (34.6)	44 (12)	87 (23.6)	12 (3.3)	119 (32.3)	(1) 0.006 (2) 0.022 (3) 0.742 (4) 0.994
2018	326 (30.7)	68 (20.9)	50 (15.3)	13 (4)	105 (32.2)	
2019	369 (34.7)	57 (15.4)	77 (20.9)	16 (4.3)	118 (32)	
**Regions**						
North	283 (26.6)	29 (10.2)	41 (14.5)	8 (2.8)	62 (21.9)	(1) 0.002 (2) 0.006 (3) 0.293 (4) <0.0001
South	780 (73.4)	140 (17.9)	173 (22.2)	33 (4.2)	280 (35.9)	
**Genders**						
Female	470 (44.2)	66 (14)	104 (22.1)	21 (4.5)	149 (31.7)	(1) 0.141 (2) 0.149 (3) 0.357 (4) 0.770
Male	593 (55.8)	103 (17.4)	110 (18.5)	20 (3.4)	193 (32.5)	
Age groups *						
≤5	136 (12.8)	24 (17.6)	62 (45.6)	12 (8.8)	74 (54.4)	(1) 0.012 (2) <0.0001 (3) 0.009 (4) <0.0001
6–15	423 (39.8)	83 (19.6)	60 (14.2)	12 (2.8)	131 (31)	
16–45	382 (35.9)	51 (13.4)	48 (12.6)	11 (2.9)	88 (23)	
≥46	122 (11.5)	11 (9)	44 (36.1)	6 (4.9)	49 (40.2)	
Clinical diagnoses **						
Non-specific febrile illness	361 (34)	49 (13.6)	70 (19.4)	14 (3.9)	105 (29.1)	(1) 0.076 (2) 0.664 (3) 0.563 (4) 0.196
Suspected Dengue	58 (5.5)	9 (15.5)	12 (20.7)	4 (6.9)	17 (29.3)	
Dengue without warning sign	604 (56.8)	108 (17.9)	121 (20)	22 (3.6)	207 (34.3)	
Dengue with warning sign	26 (2.4)	(0)	6 (23.1)	(0)	6 (23.1)	
Severe Dengue	14 (1.3)	3 (21.4)	5 (35.7)	1 (7.1)	7 (50)	
**Overall**	1063 (100)	169 (15.9)	214 (20.1)	41 (3.9)	342 (32.2)	

^+^ % in total for each variable. ^++^ % in each lane. * Age groups were defined based on the date and year of illness. ** Clinical diagnoses identified on patient’s initial hospital visit.

**Table 3:** CHIKV neutralization antibody rate. The table shows the proportion of patients with neutralizing antibodies for each factor (gender, age groups, regions, years, and clinical diagnoses). The Chi-square or Fisher’s exact test was used to compare the difference in the proportion of neutralizing antibodies between groups within each factor. The factors with statistically significant differences are analyzed in more detail in the following sections.

Variable	Patients *n* (%) ^+^	FRNT_50_ *n* (%) ^++^	*p* Value (χ² or Fisher Test)
**Gender**			
Female	470 (44.2)	54 (11.5)	0.32
Male	593 (55.8)	57 (9.6)	
**Age groups**			
≤5	136 (12.8)	19 (14)	<0.0001
6–15	423 (39.8)	34 (8)	
16–45	382 (35.9)	21 (5.5)	
≥46	122 (11.5)	37 (30.3)	
**Regions**			
North	283 (26.6)	21 (7.4)	0.052
South	780 (73.4)	90 (11.5)	
Year of illness *			
2017	368 (34.6)	39 (10.6)	0.991
2018	326 (30.7)	34 (10.4)	
2019	369 (34.7)	38 (10.3)	
Clinical diagnoses **			
**Non-specific febrile illness**	361 (34)	36 (10)	0.14
Suspected Dengue	58 (5.5)	8 (13.8)	
Dengue without warning sign	604 (56.8)	62 (10.3)	
Dengue with warning sign	26 (2.4)	1 (3.8)	
Severe Dengue	14 (1.3)	4 (28.6)	
**Overall**	1063 (100)	111 (10.4)	

^+^ % in total for each variable. ^++^ % in each lane. * Age groups were defined based on the date and year of illness. ** Clinical diagnoses identified on patient’s initial hospital visit.

**Table 4:** CHIKV RNA detection rate among 469 samples tested by RT–qPCR. The table shows the proportion of patients with RNA detection for each factor: gender, age groups, regions, years, and clinical diagnoses. The Chi-square or Fisher’s exact test was used to compare the differences in the RNA detection rate among groups for each factor.

Variable	Patients *n* (%) ^+^	RNA Detection Rate *n* (%) ^++^	*p* Value (χ² or Fisher Test)
**Genders**			
Female	209 (44.5)	52 (24.9)	0.218
Male	260 (55.4)	78 (30.0)	
**Age groups**			
≤5	90 (19.2)	20 (22.2)	0.434
6–15	187 (39.9)	55 (29.4)	
16–45	133 (28.4)	41 (30.8)	
≥46	59 (12.6)	14 (23.7)	
**Regions**			
North	66 (14.1)	13 (19.7)	0.116
South	403 (85.9)	117 (29)	
Year of illness *			
2017	149 (31.8)	42 (28.2)	0.116
2018	179 (38.2)	41 (22.9)	
2019	141 (44.6)	47 (33.3)	
Clinical diagnoses **			
Non-specific febrile illness	131 (27.9)	33 (25.2)	0.905
Suspected Dengue	27 (5.8)	9 (33.3)	
Dengue without warning sign	292 (62.3)	82 (28.1)	
Dengue with warning sign	9 (1.9)	3 (33.3)	
Severe Dengue	10 (2.1)	3 (30)	
**Overall**	469 (100)	130 (27.7)	

^+^ % in total for each variable. ^++^ % in each lane. * Age groups were defined based on the date and year of illness. ** Clinical diagnoses identified on patient’s initial hospital visit.

## Data Availability

The datasets generated and analyzed during the current study are available in the manuscript and Supplementary materials.

## Supplementary Materials

The following supporting information can be downloaded at: https://www.mdpi.com/article/10.3390/v15102065/s1, Figure S1: Neutralization antibody titer in 4 age groups in the North (A) and in the South (B); Figure S2: The rate of the anti-CHIKV IgG (A) and IgG (B) antibodies by age group in the study.; Figure S3: Detection rates of IgM and RNA in the Northern and Southern regions from 2017 to 2019; Table S1: Sample distribution in the provinces of Vietnam; Table S2: CHIKV primer used in this study.
