# Supplementary material for: Serological and Molecular Epidemiology of Chikungunya Virus Infection in Vietnam, 2017–2019
Source: Viruses. 2023 Oct 8;15(10):2065. doi: 10.3390/v15102065 (PMC10611313; doi:10.3390/v15102065)
Supplement: Supplementary file 1 [file viruses-15-02065-s001.zip › viruses-2630827-supplementary.pdf]

Article research

Serological and molecular epidemiology of Chikungunya virus infection in Vietnam, 2017 – 2019

SUPPLEMENTARY INFORMATION

Table S1: Sample distribution in the provinces of Vietnam.

Num	Region	Provinces	2017<br>n (%++)	2018<br>n (%++)	2019<br>n (%++)	Total samples<br>n (%+)
1	North	Ha Nam	115 (100)	(0)	(0)	115 (10.8)
2		Ha Noi	(0)	(0)	86 (100)	86 (8.1)
3		Hai Duong	73 (98.6)	(0)	1 (1.4)	74 (7.0)
4		Hung Yen	(0)	(0)	2 (100)	2 (0.2)
5		Bac Ninh	(0)	(0)	1 (100)	1 (0.1)
6		Hai Phong	(0)	(0)	1 (100)	1 (0.1)
7		Hoa Binh	(0)	(0)	1 (100)	1 (0.1)
8		Son La	(0)	(0)	1 (100)	1 (0.1)
9		Thai Nguyen	(0)	1 (100)	(0)	1 (0.1)
10		Thanh Hoa	(0)	1 (100)	(0)	1 (0.1)
11	South	An Giang	4 (3.3)	69 (56.6)	49 (40.2)	122 (11.5)
12		Dong Thap	32 (39)	12 (14.6)	38 (46.3)	82 (7.7)
13		Dong Nai	(0)	40 (54.1)	34 (45.9)	74 (7.0)
14		Vung Tau	36 (52.9)	30 (44.1)	2 (2.9)	68 (6.4)
15		Can Tho	18 (29.5)	42 (68.9)	1 (1.6)	61 (5.7)
16		Vinh Long	6 (10)	25 (41.7)	29 (48.3)	60 (5.6)
17		Tay Ninh	(0)	12 (26.7)	33 (73.3)	45 (4.2)
18		Long An	13 (29.5)	17 (38.6)	14 (31.8)	44 (4.1)
19		Ben Tre	1 (2.5)	1 (2.5)	38 (95)	40 (3.8)
20		Lam Dong	11 (39.3)	14 (50)	3 (10.7)	28 (2.6)
21		Soc Trang	11 (40.7)	7 (25.9)	9 (33.3)	27 (2.5)
22		Ca Mau	17 (68)	7 (28)	1 (4)	25 (2.4)
23		Hau Giang	(0)	19 (82.6)	4 (17.4)	23 (2.2)
24		Binh Phuoc	8 (42.1)	(0)	11 (57.9)	19 (1.8)
25		Binh Duong	6 (35.3)	8 (47.1)	3 (17.6)	17 (1.6)
26		Kien Giang	8 (53.3)	7 (46.7)	(0)	15 (1.4)
27		Tien Giang	1 (9.1)	7 (63.6)	3 (27.3)	11 (1.0)
28		Ho Chi Minh city	5 (71.4)	1 (14.3)	1 (14.3)	7 (0.7)
29		Tra Vinh	3 (50)	3 (50)	(0)	6 (0.6)
30		Bac Lieu	(0)	3 (60)	2 (40)	5 (0.5)
31		Phu Yen	(0)	(0)	1 (100)	1 (0.1)
**Total (%)+**			**368 (34.6)**	**326 (30.7)**	**369 (34.7)**	**1063 (100)**

Table S1 shows the distribution of residence of patients who had serum samples collected for this study. The distribution is divided into provinces according to the two regions, North and South,

and by year. The table is arranged in decreasing order of the number of serum samples collected in each province, with the percentage of samples collected compared to the total of 1063 samples shown in brackets.

+ % in total for each variable.

++ % in each lane.

Table S2: CHIKV primers used in this study. Table S2 provides detailed information about the primer pairs used in this study. The table includes the following information for each primer pair: region of gene, genome position, and nucleotide sequence of the primers [40-42].

Primer	Region	Genome position	Sequence (5′ → 3′)
**Real-time RT-PCR**			
Forward primer - P1F	E1	10364 - 10384	CTCATACCGCATCCGCATCAG
Reverse primer - P1R	E1	10470 - 10492	ACATTGGCCCCACAATGAATTTG
Forward primer – P2F	NSP2	3171 - 3194	GGCAGTGGTCCCAGATAATTCAAG
Reverse primer – P2R	NSP2	3253 - 3277	GCTGTCTAGATCCACCCCATACATG
**Conventional RT-PCR and Sequencing**			
Forward primer – P3F	E1	10246 - 10263	TACCCATTCATGTGGGGC
Reverse primer – P3R	E1	10521 - 10539	GCCTTTGTACACCACGATT

Figure S1: Neutralization antibody titer in 4 age groups in the North (A) and in the South (B). *The mean of neutralization antibody titer was compared between age groups using Kruskal-Wallis test, with Dunn-Bonferroni correction method for multiple comparison tests. P value was defined as follows * p≤0.05; ** p≤0.01; **** p<0.0001.*

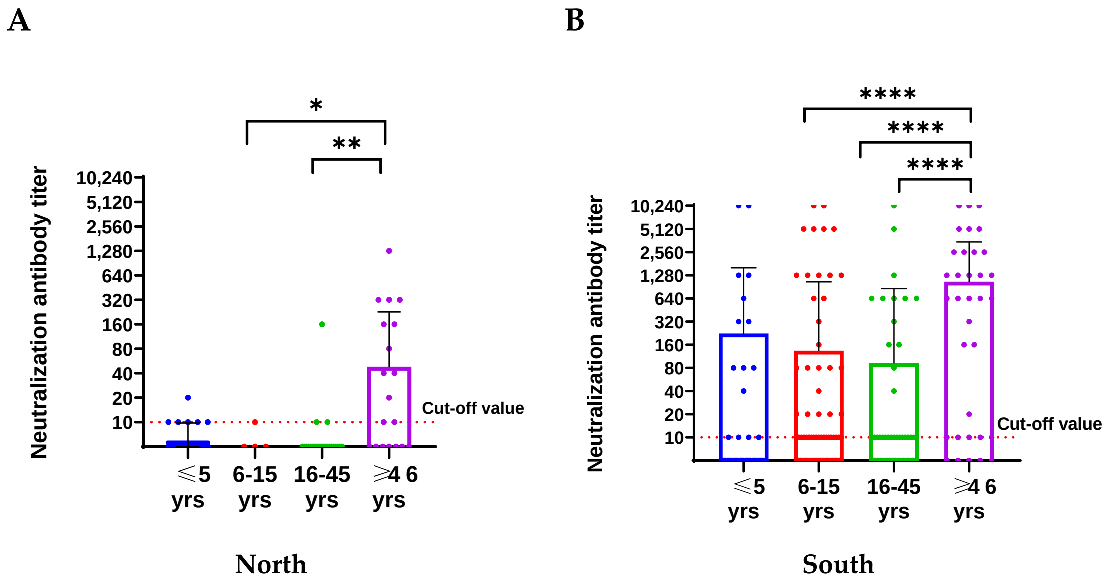

Figure S2: The rate of the anti-CHIKV *IgG (A) and IgG (B) antibodies by age group in the study.*

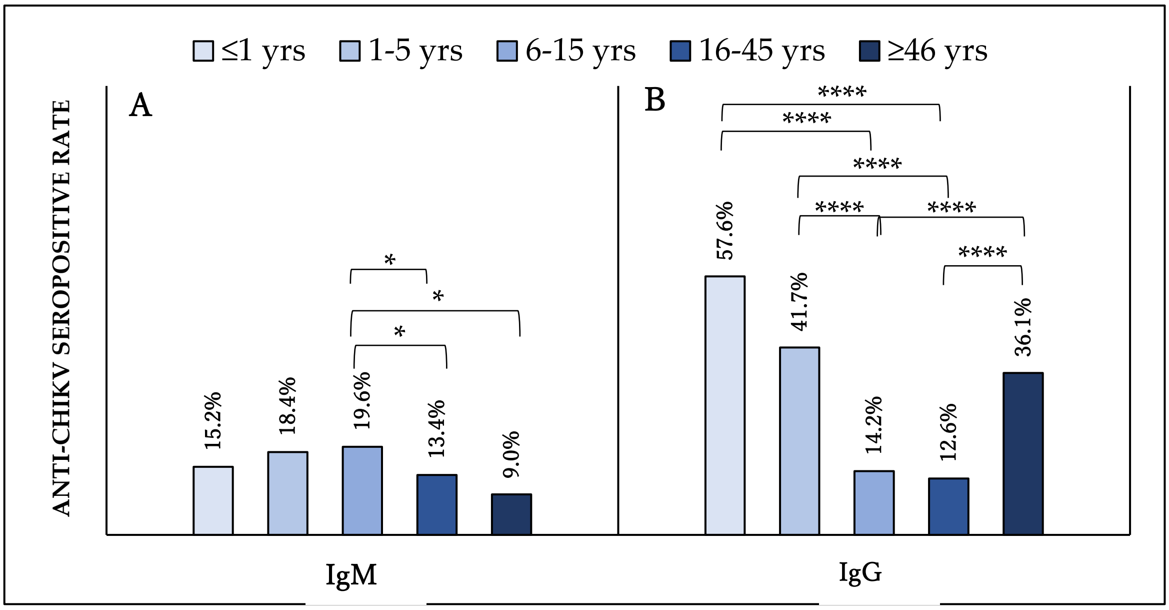

Figure S2 shows the distribution of IgM (A) and IgM (B) antibodies in different age groups. The color scheme of light blue to dark blue indicates the following age groups: under 1 year old, 1- to 5-year-olds, 6- to 15-year-olds, 16- to 45-year-olds, and over 46-year-olds. IgM and IgG rate between age groups were compared using the Generalized Linear Model (GLM). P *value was defined as follows * $p \leq 0.05$; and **** $p < 0.0001$.*

Figure S3: Detection rates of IgM and RNA in the Northern and Southern regions from 2017 to 2019

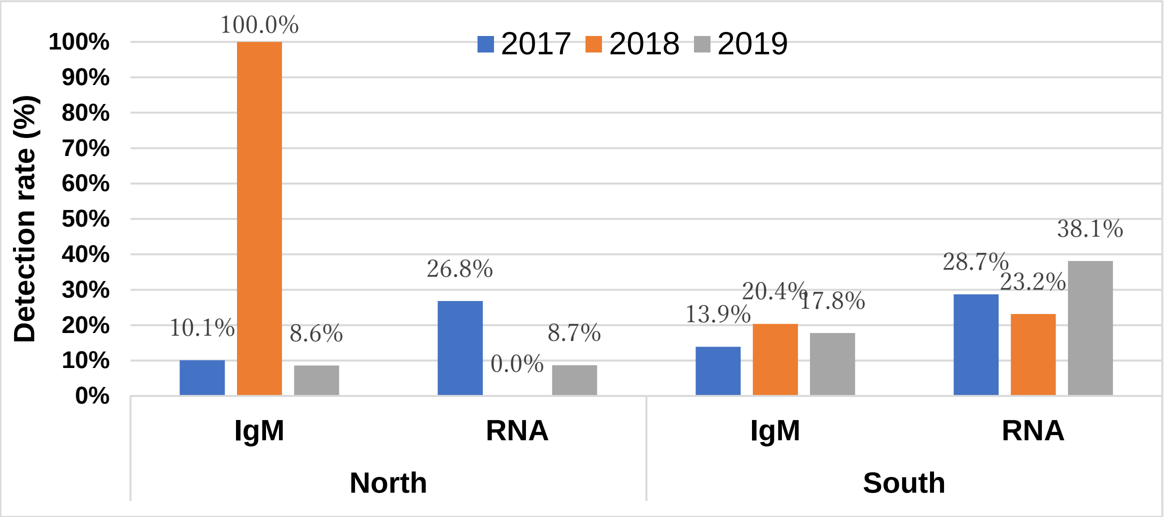

Figure S3 shows the detection rates of IgM and RNA in the Northern and Southern regions over the years 2017 (blue), 2018 (orange), and 2019 (gray). The Generalized Linear Model (GLM) was used to compare detection rates between years in each region. The results showed that there was no statistically significant difference in detection rates between years in either region ($p > 0.05$).
